# *N*′-[(*E*)-1-(2-Hy­droxy­phen­yl)ethyl­idene]pyrazine-2-carbohydrazide

**DOI:** 10.1107/S1600536813022137

**Published:** 2013-08-14

**Authors:** Shahid Hameed, Mushtaq Ahmad, M. Nawaz Tahir, Muhammad Israr, Muhammad Anwar

**Affiliations:** a Department of Chemistry, Quaid-i-Azam University, Islamabad, Pakistan; b Medicinal Botanic Centre, PCSIR Laboratories Complex, Peshawar, Pakistan; c University of Sargodha, Department of Physics, Sargodha, Pakistan; d Department of Chemistry, Kohat University of Science and Technology, Kohat, Pakistan

## Abstract

The title compound, C_13_H_12_N_4_O_2_, crystallized with two independent mol­ecules (*A* and *B*) in the asymmetric unit. Mol­ecule *B* is planar to within 0.044 (3) Å for all non-H atoms, while mol­ecule *A* is slightly twisted, with a dihedral angle of 6.29 (4)° between the mean planes of the pyrazine-2-carbohydrazide and 1-(2-hy­droxy­phen­yl)ethanone moieties (r.m.s. deviations = 0.0348 and 0.0428 Å, respectively). *S*(5) and *S*(6) ring motifs are formed in both mol­ecules due to the presence of intra­molecular O—H⋯N and N—H⋯N hydrogen bonds. In the crystal, mol­ecules *A* and *B* are linked by a C—H⋯O hydrogen bond. They stack along the *a*-axis direction, forming columns with π–π inter­actions involving inversion-related pyrazine and benzene rings [centroid–centroid distances = 3.5489 (13)–3.8513 (16) Å].

## Related literature

For a related crystal structure and other studies, see: Hameed *et al.* (2013 ▶). For graph-set notation, see: Bernstein *et al.* (1995 ▶).
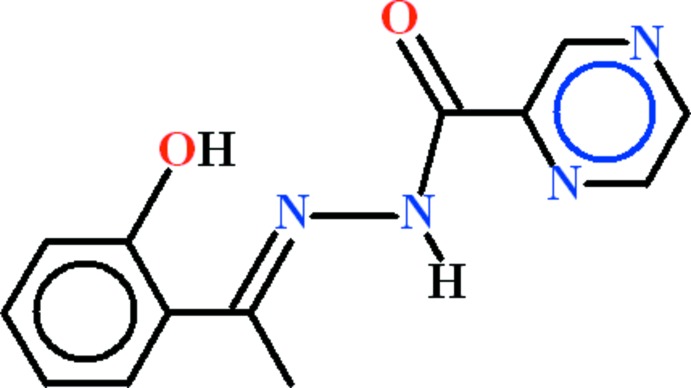


## Experimental

### 

#### Crystal data

C_13_H_12_N_4_O_2_
*M* *_r_* = 256.27
Triclinic, 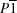

*a* = 7.1767 (7) Å
*b* = 10.1743 (10) Å
*c* = 17.1150 (17) Å
α = 86.172 (3)°
β = 85.275 (2)°
γ = 80.963 (4)°
*V* = 1228.2 (2) Å^3^
*Z* = 4
Mo *K*α radiation
μ = 0.10 mm^−1^
*T* = 296 K
0.28 × 0.23 × 0.20 mm

#### Data collection

Bruker Kappa APEXII CCD diffractometer
Absorption correction: multi-scan (*SADABS*; Bruker, 2005 ▶) *T*_min_ = 0.973, *T*_max_ = 0.981
19042 measured reflections
4821 independent reflections
2608 reflections with *I* > 2σ(*I*)
*R*_int_ = 0.048

#### Refinement

*R*[*F* ^2^ > 2σ(*F* ^2^)] = 0.050
*wR*(*F* ^2^) = 0.138
*S* = 1.00
4821 reflections
361 parameters
H atoms treated by a mixture of independent and constrained refinement
Δρ_max_ = 0.23 e Å^−3^
Δρ_min_ = −0.16 e Å^−3^

### 

Data collection: *APEX2* (Bruker, 2007 ▶); cell refinement: *SAINT* (Bruker, 2007 ▶); data reduction: *SAINT*; program(s) used to solve structure: *SHELXS97* (Sheldrick, 2008 ▶); program(s) used to refine structure: *SHELXL97* (Sheldrick, 2008 ▶); molecular graphics: *ORTEP-3 for Windows* (Farrugia, 2012 ▶) and *PLATON* (Spek, 2009 ▶); software used to prepare material for publication: *WinGX* (Farrugia, 2012 ▶) and *PLATON* (Spek, 2009 ▶).

## Supplementary Material

Crystal structure: contains datablock(s) global, I. DOI: 10.1107/S1600536813022137/su2634sup1.cif

Structure factors: contains datablock(s) I. DOI: 10.1107/S1600536813022137/su2634Isup2.hkl

Supplementary material file. DOI: 10.1107/S1600536813022137/su2634Isup3.cml

Additional supplementary materials:  crystallographic information; 3D view; checkCIF report

## Figures and Tables

**Table 1:** Hydrogen-bond geometry (Å, °)

*D*—H⋯*A*	*D*—H	H⋯*A*	*D*⋯*A*	*D*—H⋯*A*
O1—H1⋯N1	0.82	1.82	2.537 (2)	145
N2—H2*A*⋯N3	0.80 (2)	2.26 (2)	2.654 (3)	111.5 (17)
O3—H3*A*⋯N5	0.82	1.82	2.534 (3)	145
N6—H6⋯N7	0.80 (3)	2.21 (3)	2.628 (3)	113 (3)
C3—H3⋯O3^i^	0.93	2.59	3.403 (3)	146

Symmetry code: (i) 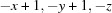.

**Fig. 1.**
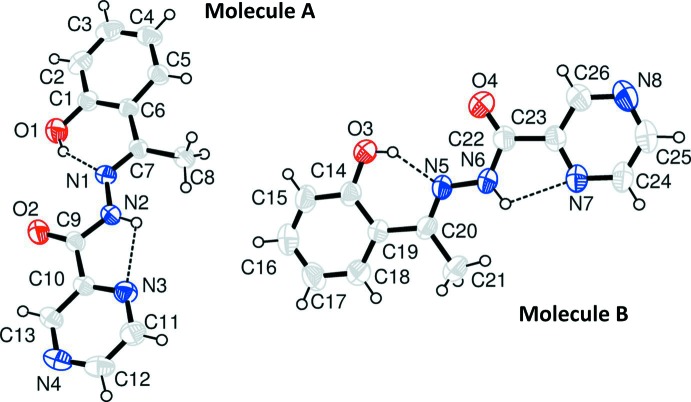
View of the molecular structure of the two independent molecules (A and B) of the title compound, with atom labelling. Displacement ellipsoids are drawn at the 50% probability level. The intramolecular hydrogen bonds are shown as dashed lines (see Table 1 for details).

**Fig. 2.**
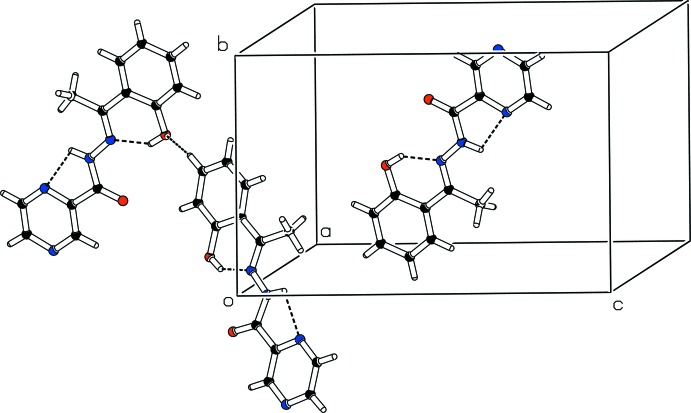
The crystal packing diagram of the title compound, viewed along the a axis. The various hydrogen bonds are shown as dashed lines (see Table 1 for details).

## supplementary crystallographic information

### 1. Comment

The title compound was prepared in continuation of our interest in the synthesis of compounds containing the moiety pyrazine-2-carbohydrazide (Hameed *et al.*, 2013).

The title compound crystallized with two independent molecules (A and B) in the asymmetric unit, Fig. 1. In molecule A, the 1-(2-hydroxyphenyl)ethanone (C1—C8/O1) moiety and the pyrazine-2-carbohydrazide moiety (C9–C13/N1–N4/O2) are almost planar with r.m.s. deviations of 0.0348 Å and 0.0428 Å, respectively. They are inclined to one another by 6.289 (44)°. In molecule B, similar groups (C14—C21/O3) and (C22—C26/N5—N8/O4) are planar with r.m.s. deviations of 0.0111 and 0.019 Å, respectively, and are inclined to one another by only 0.305 (21)° *i.e.* almost coplanar.

There exist strong intramolecular N—H···N and O—H···N hydrogen bonds in each molecule (Table 1 and Fig. 1) forming S(5) and S(6) ring motifs (Bernstein *et al.*, 1995).

In the crystal, molecules A and B are linked by a C—H···O hydrogen bond (Table 1 and Fig. 2). They stack along the a axis direction forming columns with π–π interactions involving inversion related pyrazine and benzene rings. The centroid-to-centroid distances are 3.5489 (13) Å [*Cg*1—*Cg*2^i^], 3.6289 (13) Å [*Cg*1—*Cg*2^ii^], 3.7738 (16) Å [*Cg*3—*Cg*4^iii^], and 3.8513 (16) Å [*Cg*3—*Cg*4^iv^], where Cg1, Cg2, Cg3 and Cg4 are the centroids of rings (C10/N3/C11/C12/N4/C13), (C1—C6), (C23/N7/C24/C25/N8/C26) and (C14—C19), respectively [symmetry codes: (i) = -*x*, - *y*, -*z;* (ii) = - *x*+1, - *y*, -*z;* (iii) = -*x*, - *y*+1, - *z*+1; (iv) = - *x*+1, - *y*+1, - *z+1]*.

### 2. Experimental

The title compound was prepared by the condensation of an equimolar ratio of pyrazine-2-carbohydrazide (0.50 g, 3.6 mmol) and 1-(2-hydroxyphenyl)ethanone (0.45 ml, 3.6 mmol) in methanol by stirring well and then refluxing of 5 h. The resulting reaction mixture was allowed to cool over night. The precipitated solid was filtered, washed with petroleum ether and recrystallized from chloroform in petroleum ether and then dried under reduced pressure over CaCl_2_ to give colourless prisms.

### 3. Refinement

The H-atom of the amide and one of the methyl groups were refined with U_iso_(H) = 1.2U_eq_(N) and = 1.5U_eq_(C-methyl). The other H-atoms were positioned geometrically (C–H = 0.93 - 0.96 Å, O–H = 0.82 Å) and refined as riding with *U*_iso_(H) = x × U_eq_(C,N,O), where x = 1.5 for hydroxy and methyl H atoms and = 1.2 for other H atoms.

### Crystal data

**Table:**

C_13_H_12_N_4_O_2_	*Z* = 4
*M**_r_* = 256.27	*F*(000) = 536
Triclinic, *P*1	*D*_x_ = 1.386 Mg m^−^^3^
Hall symbol: -P 1	Mo *K*α radiation, λ = 0.71073 Å
*a* = 7.1767 (7) Å	Cell parameters from 2608 reflections
*b* = 10.1743 (10) Å	θ = 1.2–26.0°
*c* = 17.1150 (17) Å	µ = 0.10 mm^−^^1^
α = 86.172 (3)°	*T* = 296 K
β = 85.275 (2)°	Prism, colourless
γ = 80.963 (4)°	0.28 × 0.23 × 0.20 mm
*V* = 1228.2 (2) Å^3^	

### Data collection

**Table:**

Bruker Kappa APEXII CCD diffractometer	4821 independent reflections
Radiation source: fine-focus sealed tube	2608 reflections with *I* > 2σ(*I*)
Graphite monochromator	*R*_int_ = 0.048
Detector resolution: 8.00 pixels mm^-1^	θ_max_ = 26.0°, θ_min_ = 1.2°
ω scans	*h* = −8→8
Absorption correction: multi-scan (*SADABS*; Bruker, 2005)	*k* = −12→12
*T*_min_ = 0.973, *T*_max_ = 0.981	*l* = −21→21
19042 measured reflections	

### Refinement

**Table:**

Refinement on *F*^2^	Secondary atom site location: difference Fourier map
Least-squares matrix: full	Hydrogen site location: inferred from neighbouring sites
*R*[*F*^2^ > 2σ(*F*^2^)] = 0.050	H atoms treated by a mixture of independent and constrained refinement
*wR*(*F*^2^) = 0.138	*w* = 1/[σ^2^(*F*_o_^2^) + (0.0537*P*)^2^ + 0.2363*P*] where *P* = (*F*_o_^2^ + 2*F*_c_^2^)/3
*S* = 1.00	(Δ/σ)_max_ < 0.001
4821 reflections	Δρ_max_ = 0.23 e Å^−^^3^
361 parameters	Δρ_min_ = −0.16 e Å^−^^3^
0 restraints	Extinction correction: *SHELXL97* (Sheldrick, 2008), Fc^*^=kFc[1+0.001xFc^2^λ^3^/sin(2θ)]^-1/4^
Primary atom site location: structure-invariant direct methods	Extinction coefficient: 0.0083 (12)

### Special details

**Table:**

Geometry. Bond distances, angles etc. have been calculated using the rounded fractional coordinates. All su's are estimated from the variances of the (full) variance-covariance matrix. The cell esds are taken into account in the estimation of distances, angles and torsion angles
Refinement. Refinement of *F*^2^ against ALL reflections. The weighted *R*-factor *wR* and goodness of fit *S* are based on *F*^2^, conventional *R*-factors *R* are based on *F*, with *F* set to zero for negative *F*^2^. The threshold expression of *F*^2^ > σ(*F*^2^) is used only for calculating *R*-factors(gt) *etc*. and is not relevant to the choice of reflections for refinement. *R*-factors based on *F*^2^ are statistically about twice as large as those based on *F*, and *R*-factors based on ALL data will be even larger.

### Fractional atomic coordinates and isotropic or equivalent isotropic displacement parameters (Å^2^)

**Table:**

	*x*	*y*	*z*	*U*_iso_*/*U*_eq_	
O1	0.4150 (3)	0.07549 (16)	−0.16151 (9)	0.0602 (7)	
O2	0.2080 (2)	−0.19271 (16)	−0.05633 (10)	0.0582 (6)	
N1	0.2781 (2)	0.04641 (18)	−0.02111 (10)	0.0372 (6)	
N2	0.2155 (3)	−0.04221 (18)	0.03446 (11)	0.0398 (7)	
N3	0.0878 (2)	−0.20255 (18)	0.14825 (12)	0.0456 (7)	
N4	0.0423 (3)	−0.46305 (19)	0.11955 (14)	0.0545 (8)	
C1	0.4198 (3)	0.2026 (2)	−0.14404 (14)	0.0421 (8)	
C2	0.4732 (3)	0.2888 (3)	−0.20396 (15)	0.0561 (10)	
C3	0.4803 (3)	0.4192 (3)	−0.19149 (17)	0.0604 (11)	
C4	0.4368 (3)	0.4658 (2)	−0.11752 (17)	0.0560 (10)	
C5	0.3875 (3)	0.3803 (2)	−0.05687 (14)	0.0448 (8)	
C6	0.3735 (3)	0.2475 (2)	−0.06741 (13)	0.0358 (7)	
C7	0.3094 (3)	0.1610 (2)	−0.00190 (12)	0.0352 (7)	
C8	0.2827 (3)	0.2063 (2)	0.08019 (13)	0.0515 (9)	
C9	0.1834 (3)	−0.1606 (2)	0.01156 (14)	0.0382 (8)	
C10	0.1186 (3)	−0.2501 (2)	0.07671 (13)	0.0371 (8)	
C11	0.0343 (3)	−0.2871 (3)	0.20495 (16)	0.0561 (10)	
C12	0.0136 (3)	−0.4158 (3)	0.19042 (17)	0.0573 (10)	
C13	0.0928 (3)	−0.3772 (2)	0.06276 (15)	0.0469 (9)	
O3	0.2432 (3)	0.48626 (18)	0.34993 (10)	0.0754 (8)	
O4	0.1729 (3)	0.7739 (2)	0.48091 (12)	0.0870 (9)	
N5	0.2460 (3)	0.5031 (2)	0.49687 (11)	0.0491 (7)	
N6	0.2206 (3)	0.5847 (2)	0.55841 (12)	0.0557 (8)	
N7	0.1834 (3)	0.7165 (2)	0.68710 (12)	0.0550 (8)	
N8	0.0968 (3)	0.9941 (2)	0.68391 (16)	0.0754 (10)	
C14	0.2858 (3)	0.3535 (3)	0.36491 (14)	0.0500 (9)	
C15	0.3115 (4)	0.2738 (3)	0.30127 (15)	0.0617 (11)	
C16	0.3510 (4)	0.1389 (3)	0.31072 (17)	0.0671 (11)	
C17	0.3662 (4)	0.0795 (3)	0.38440 (19)	0.0747 (11)	
C18	0.3421 (4)	0.1569 (3)	0.44851 (16)	0.0682 (11)	
C19	0.3018 (3)	0.2958 (2)	0.44095 (13)	0.0454 (8)	
C20	0.2773 (3)	0.3760 (3)	0.51033 (13)	0.0496 (9)	
C21	0.2897 (7)	0.3111 (4)	0.5907 (2)	0.104 (2)	
C22	0.1843 (4)	0.7184 (3)	0.54528 (16)	0.0556 (10)	
C23	0.1605 (3)	0.7897 (3)	0.62001 (15)	0.0502 (9)	
C24	0.1623 (4)	0.7838 (3)	0.75194 (15)	0.0633 (10)	
C25	0.1216 (4)	0.9200 (3)	0.74989 (18)	0.0689 (11)	
C26	0.1158 (4)	0.9267 (3)	0.61878 (17)	0.0693 (11)	
H1	0.36890	0.03550	−0.12340	0.0900*	
H2	0.50520	0.25780	−0.25390	0.0670*	
H2A	0.202 (3)	−0.031 (2)	0.0805 (13)	0.0480*	
H3	0.51450	0.47650	−0.23300	0.0720*	
H4	0.44080	0.55450	−0.10880	0.0670*	
H5	0.36250	0.41190	−0.00680	0.0540*	
H8A	0.27220	0.13110	0.11640	0.0770*	
H8B	0.16950	0.27020	0.08570	0.0770*	
H8C	0.38940	0.24660	0.09110	0.0770*	
H11	0.01000	−0.25890	0.25590	0.0670*	
H12	−0.02200	−0.47180	0.23230	0.0690*	
H13	0.11150	−0.40420	0.01150	0.0560*	
H3A	0.23690	0.52440	0.39100	0.1130*	
H6	0.234 (4)	0.557 (3)	0.6029 (15)	0.0670*	
H15	0.30140	0.31340	0.25090	0.0740*	
H16	0.36760	0.08720	0.26720	0.0810*	
H17	0.39280	−0.01280	0.39120	0.0900*	
H18	0.35300	0.11540	0.49840	0.0820*	
H21A	0.250 (6)	0.367 (4)	0.628 (3)	0.1560*	
H21B	0.409 (6)	0.269 (4)	0.598 (2)	0.1560*	
H21C	0.210 (6)	0.246 (4)	0.597 (2)	0.1560*	
H24	0.17570	0.73700	0.80020	0.0760*	
H25	0.11100	0.96230	0.79690	0.0820*	
H26	0.09830	0.97380	0.57080	0.0830*	

### Atomic displacement parameters (Å^2^)

**Table:**

	*U*^11^	*U*^22^	*U*^33^	*U*^12^	*U*^13^	*U*^23^
O1	0.0827 (13)	0.0529 (11)	0.0456 (11)	−0.0117 (9)	0.0008 (9)	−0.0108 (9)
O2	0.0818 (12)	0.0506 (10)	0.0459 (11)	−0.0196 (9)	−0.0032 (9)	−0.0089 (9)
N1	0.0369 (10)	0.0324 (10)	0.0419 (11)	−0.0046 (8)	−0.0035 (8)	0.0004 (9)
N2	0.0458 (11)	0.0354 (11)	0.0388 (12)	−0.0082 (8)	−0.0029 (9)	−0.0019 (10)
N3	0.0471 (11)	0.0425 (12)	0.0472 (13)	−0.0096 (9)	−0.0004 (9)	−0.0001 (10)
N4	0.0479 (12)	0.0366 (12)	0.0775 (17)	−0.0067 (9)	−0.0032 (11)	0.0063 (12)
C1	0.0404 (13)	0.0423 (14)	0.0440 (15)	−0.0061 (10)	−0.0073 (10)	−0.0003 (12)
C2	0.0533 (16)	0.0713 (19)	0.0426 (16)	−0.0109 (13)	−0.0040 (12)	0.0079 (14)
C3	0.0508 (16)	0.066 (2)	0.066 (2)	−0.0227 (13)	−0.0113 (14)	0.0253 (16)
C4	0.0527 (15)	0.0438 (15)	0.075 (2)	−0.0178 (12)	−0.0159 (14)	0.0102 (15)
C5	0.0430 (13)	0.0397 (14)	0.0530 (16)	−0.0082 (10)	−0.0080 (11)	−0.0020 (12)
C6	0.0310 (11)	0.0341 (13)	0.0418 (14)	−0.0024 (9)	−0.0072 (9)	0.0012 (10)
C7	0.0321 (11)	0.0322 (13)	0.0409 (14)	−0.0008 (9)	−0.0061 (9)	−0.0049 (10)
C8	0.0696 (16)	0.0409 (14)	0.0450 (15)	−0.0124 (12)	−0.0007 (12)	−0.0045 (12)
C9	0.0372 (12)	0.0332 (13)	0.0449 (15)	−0.0032 (10)	−0.0080 (10)	−0.0054 (11)
C10	0.0305 (11)	0.0331 (13)	0.0468 (15)	−0.0021 (9)	−0.0048 (10)	−0.0005 (11)
C11	0.0584 (16)	0.0580 (17)	0.0517 (17)	−0.0135 (13)	0.0022 (12)	0.0015 (14)
C12	0.0470 (15)	0.0517 (17)	0.071 (2)	−0.0116 (12)	0.0003 (13)	0.0166 (15)
C13	0.0451 (14)	0.0353 (14)	0.0600 (17)	−0.0040 (11)	−0.0044 (12)	−0.0044 (13)
O3	0.1333 (18)	0.0556 (12)	0.0381 (11)	−0.0212 (11)	0.0008 (11)	−0.0001 (9)
O4	0.148 (2)	0.0666 (13)	0.0462 (13)	−0.0209 (13)	−0.0017 (12)	0.0016 (11)
N5	0.0622 (13)	0.0506 (13)	0.0357 (12)	−0.0122 (10)	0.0009 (9)	−0.0085 (10)
N6	0.0763 (15)	0.0575 (15)	0.0338 (12)	−0.0110 (11)	−0.0004 (11)	−0.0090 (12)
N7	0.0584 (13)	0.0605 (14)	0.0467 (14)	−0.0059 (10)	−0.0033 (10)	−0.0146 (12)
N8	0.0934 (19)	0.0602 (16)	0.0776 (19)	−0.0186 (13)	−0.0074 (15)	−0.0238 (15)
C14	0.0542 (15)	0.0568 (17)	0.0399 (15)	−0.0139 (12)	0.0040 (11)	−0.0066 (13)
C15	0.0750 (19)	0.071 (2)	0.0413 (16)	−0.0182 (15)	0.0036 (13)	−0.0133 (14)
C16	0.0650 (18)	0.080 (2)	0.058 (2)	−0.0068 (15)	−0.0027 (14)	−0.0279 (17)
C17	0.092 (2)	0.0533 (17)	0.077 (2)	0.0073 (15)	−0.0161 (17)	−0.0223 (17)
C18	0.087 (2)	0.0600 (19)	0.0557 (18)	−0.0001 (15)	−0.0147 (15)	−0.0041 (15)
C19	0.0470 (14)	0.0497 (15)	0.0397 (15)	−0.0068 (11)	−0.0023 (11)	−0.0055 (12)
C20	0.0567 (15)	0.0554 (17)	0.0361 (14)	−0.0074 (12)	−0.0025 (11)	−0.0026 (12)
C21	0.194 (5)	0.072 (3)	0.0387 (19)	0.002 (3)	−0.011 (2)	0.0014 (17)
C22	0.0675 (17)	0.0532 (18)	0.0481 (18)	−0.0152 (13)	−0.0005 (13)	−0.0078 (14)
C23	0.0532 (15)	0.0532 (16)	0.0468 (17)	−0.0140 (12)	−0.0013 (12)	−0.0101 (13)
C24	0.0668 (17)	0.075 (2)	0.0491 (17)	−0.0044 (14)	−0.0086 (13)	−0.0206 (15)
C25	0.0684 (19)	0.076 (2)	0.066 (2)	−0.0119 (16)	−0.0047 (15)	−0.0304 (18)
C26	0.091 (2)	0.0570 (19)	0.063 (2)	−0.0200 (15)	−0.0050 (15)	−0.0071 (16)

### Geometric parameters (Å, º)

**Table:**

O1—C1	1.353 (3)	C11—C12	1.382 (4)
O2—C9	1.219 (3)	C2—H2	0.9300
O1—H1	0.8200	C3—H3	0.9300
O3—C14	1.349 (3)	C4—H4	0.9300
O4—C22	1.207 (3)	C5—H5	0.9300
O3—H3A	0.8200	C8—H8B	0.9600
N1—N2	1.364 (3)	C8—H8C	0.9600
N1—C7	1.290 (3)	C8—H8A	0.9600
N2—C9	1.350 (3)	C11—H11	0.9300
N3—C11	1.327 (3)	C12—H12	0.9300
N3—C10	1.335 (3)	C13—H13	0.9300
N4—C13	1.329 (3)	C14—C15	1.384 (4)
N4—C12	1.323 (4)	C14—C19	1.398 (3)
N2—H2A	0.80 (2)	C15—C16	1.359 (4)
N5—N6	1.367 (3)	C16—C17	1.367 (4)
N5—C20	1.286 (4)	C17—C18	1.377 (4)
N6—C22	1.351 (4)	C18—C19	1.397 (4)
N7—C24	1.328 (3)	C19—C20	1.469 (3)
N7—C23	1.334 (3)	C20—C21	1.489 (4)
N8—C25	1.324 (4)	C22—C23	1.497 (4)
N8—C26	1.334 (4)	C23—C26	1.380 (4)
N6—H6	0.80 (3)	C24—C25	1.369 (4)
C1—C6	1.414 (3)	C15—H15	0.9300
C1—C2	1.374 (4)	C16—H16	0.9300
C2—C3	1.367 (4)	C17—H17	0.9300
C3—C4	1.376 (4)	C18—H18	0.9300
C4—C5	1.369 (3)	C21—H21A	0.88 (5)
C5—C6	1.395 (3)	C21—H21B	0.91 (4)
C6—C7	1.468 (3)	C21—H21C	0.94 (4)
C7—C8	1.496 (3)	C24—H24	0.9300
C9—C10	1.484 (3)	C25—H25	0.9300
C10—C13	1.374 (3)	C26—H26	0.9300
			
C1—O1—H1	109.00	C7—C8—H8A	109.00
C14—O3—H3A	110.00	N3—C11—H11	119.00
N2—N1—C7	120.53 (18)	C12—C11—H11	119.00
N1—N2—C9	118.47 (18)	N4—C12—H12	118.00
C10—N3—C11	115.5 (2)	C11—C12—H12	119.00
C12—N4—C13	115.0 (2)	C10—C13—H13	119.00
C9—N2—H2A	116.9 (15)	N4—C13—H13	119.00
N1—N2—H2A	124.6 (15)	C15—C14—C19	120.1 (3)
N6—N5—C20	119.58 (19)	O3—C14—C15	117.3 (2)
N5—N6—C22	120.3 (2)	O3—C14—C19	122.6 (2)
C23—N7—C24	115.9 (2)	C14—C15—C16	121.5 (3)
C25—N8—C26	115.2 (2)	C15—C16—C17	119.8 (3)
C22—N6—H6	117 (2)	C16—C17—C18	119.7 (3)
N5—N6—H6	123 (2)	C17—C18—C19	122.0 (3)
O1—C1—C2	117.5 (2)	C18—C19—C20	120.9 (2)
C2—C1—C6	120.1 (2)	C14—C19—C18	116.9 (2)
O1—C1—C6	122.37 (19)	C14—C19—C20	122.2 (2)
C1—C2—C3	121.3 (2)	N5—C20—C21	123.2 (3)
C2—C3—C4	120.0 (3)	N5—C20—C19	116.0 (2)
C3—C4—C5	119.4 (2)	C19—C20—C21	120.8 (3)
C4—C5—C6	122.5 (2)	O4—C22—N6	124.0 (3)
C1—C6—C5	116.7 (2)	O4—C22—C23	123.9 (3)
C1—C6—C7	122.37 (18)	N6—C22—C23	112.0 (2)
C5—C6—C7	120.9 (2)	C22—C23—C26	120.6 (2)
N1—C7—C6	115.00 (18)	N7—C23—C22	117.8 (3)
C6—C7—C8	121.04 (18)	N7—C23—C26	121.6 (2)
N1—C7—C8	123.96 (19)	N7—C24—C25	122.0 (3)
O2—C9—N2	123.4 (2)	N8—C25—C24	122.9 (3)
O2—C9—C10	122.60 (19)	N8—C26—C23	122.4 (3)
N2—C9—C10	114.0 (2)	C14—C15—H15	119.00
N3—C10—C13	122.1 (2)	C16—C15—H15	119.00
C9—C10—C13	120.3 (2)	C15—C16—H16	120.00
N3—C10—C9	117.61 (18)	C17—C16—H16	120.00
N3—C11—C12	121.8 (3)	C16—C17—H17	120.00
N4—C12—C11	122.9 (3)	C18—C17—H17	120.00
N4—C13—C10	122.7 (2)	C17—C18—H18	119.00
C3—C2—H2	119.00	C19—C18—H18	119.00
C1—C2—H2	119.00	C20—C21—H21A	113 (3)
C2—C3—H3	120.00	C20—C21—H21B	111 (2)
C4—C3—H3	120.00	C20—C21—H21C	109 (2)
C3—C4—H4	120.00	H21A—C21—H21B	111 (4)
C5—C4—H4	120.00	H21A—C21—H21C	106 (4)
C4—C5—H5	119.00	H21B—C21—H21C	107 (4)
C6—C5—H5	119.00	N7—C24—H24	119.00
H8A—C8—H8B	110.00	C25—C24—H24	119.00
H8A—C8—H8C	109.00	N8—C25—H25	119.00
C7—C8—H8C	109.00	C24—C25—H25	119.00
C7—C8—H8B	109.00	N8—C26—H26	119.00
H8B—C8—H8C	110.00	C23—C26—H26	119.00
			
C7—N1—N2—C9	179.6 (2)	C1—C6—C7—N1	−7.5 (3)
N2—N1—C7—C6	−179.11 (18)	C1—C6—C7—C8	173.0 (2)
N2—N1—C7—C8	0.5 (3)	C5—C6—C7—C8	−8.5 (3)
N1—N2—C9—O2	0.2 (3)	N2—C9—C10—N3	4.2 (3)
N1—N2—C9—C10	179.11 (18)	N2—C9—C10—C13	−175.6 (2)
C11—N3—C10—C9	−178.49 (19)	O2—C9—C10—N3	−177.0 (2)
C11—N3—C10—C13	1.2 (3)	O2—C9—C10—C13	3.3 (3)
C10—N3—C11—C12	0.5 (3)	N3—C10—C13—N4	−2.5 (3)
C13—N4—C12—C11	0.0 (3)	C9—C10—C13—N4	177.2 (2)
C12—N4—C13—C10	1.8 (3)	N3—C11—C12—N4	−1.1 (4)
C20—N5—N6—C22	−178.8 (2)	O3—C14—C15—C16	−178.7 (3)
N6—N5—C20—C19	179.7 (2)	C19—C14—C15—C16	0.5 (4)
N6—N5—C20—C21	−0.7 (4)	O3—C14—C19—C18	178.5 (2)
N5—N6—C22—O4	−0.4 (4)	O3—C14—C19—C20	−1.3 (3)
N5—N6—C22—C23	179.8 (2)	C15—C14—C19—C18	−0.6 (3)
C24—N7—C23—C22	179.6 (2)	C15—C14—C19—C20	179.5 (2)
C24—N7—C23—C26	−0.6 (4)	C14—C15—C16—C17	−0.1 (4)
C23—N7—C24—C25	−0.7 (4)	C15—C16—C17—C18	−0.3 (4)
C26—N8—C25—C24	−0.5 (4)	C16—C17—C18—C19	0.1 (4)
C25—N8—C26—C23	−0.8 (4)	C17—C18—C19—C14	0.3 (4)
O1—C1—C6—C5	178.8 (2)	C17—C18—C19—C20	−179.8 (2)
O1—C1—C6—C7	−2.6 (3)	C14—C19—C20—N5	−2.1 (3)
C2—C1—C6—C7	177.9 (2)	C14—C19—C20—C21	178.2 (3)
O1—C1—C2—C3	179.5 (2)	C18—C19—C20—N5	178.1 (2)
C2—C1—C6—C5	−0.8 (3)	C18—C19—C20—C21	−1.7 (4)
C6—C1—C2—C3	−0.9 (3)	O4—C22—C23—N7	−177.6 (3)
C1—C2—C3—C4	1.2 (4)	O4—C22—C23—C26	2.6 (4)
C2—C3—C4—C5	0.3 (3)	N6—C22—C23—N7	2.2 (3)
C3—C4—C5—C6	−2.1 (3)	N6—C22—C23—C26	−177.6 (2)
C4—C5—C6—C7	−176.4 (2)	N7—C23—C26—N8	1.4 (4)
C4—C5—C6—C1	2.3 (3)	C22—C23—C26—N8	−178.8 (2)
C5—C6—C7—N1	171.1 (2)	N7—C24—C25—N8	1.3 (5)

### Hydrogen-bond geometry (Å, º)

**Table:**

*D*—H···*A*	*D*—H	H···*A*	*D*···*A*	*D*—H···*A*
O1—H1···N1	0.82	1.82	2.537 (2)	145
N2—H2*A*···N3	0.80 (2)	2.26 (2)	2.654 (3)	111.5 (17)
O3—H3*A*···N5	0.82	1.82	2.534 (3)	145
N6—H6···N7	0.80 (3)	2.21 (3)	2.628 (3)	113 (3)
C3—H3···O3^i^	0.93	2.59	3.403 (3)	146

Symmetry code: (i) −*x*+1, −*y*+1, −*z*.
